# Stakeholder perceptions and experiences from the implementation of the Gratuité user fee exemption policy in Burkina Faso: a qualitative study

**DOI:** 10.1186/s12961-023-01008-3

**Published:** 2023-06-06

**Authors:** Aduragbemi Banke-Thomas, Marie-Jeanne Offosse, Pierre Yameogo, Astrid Raissa Manli, Aude Goumbri, Cephas Avoka, Matt Boxshall, Ejemai Eboreime

**Affiliations:** 1 ThinkWell Institute, 11 B.P. 1255 CMS 11 Ouagadougou, Quartier Ouaga 2000, près de la fondation Kimi, à 500 du boulevard Muammar Kadaffi, Ouagadougou, Burkina Faso; 2 Faculty of Epidemiology and Population Health, London School of Hygiene and Tropical Medicine, London, United Kingdom; 3 School of Human Sciences, University of Greenwich, London, United Kingdom; 4 Ministry of Health, Ouagadougou, Burkina Faso; 5 Department of Psychiatry, Faculty of Medicine & Dentistry, University of Alberta, Edmonton, Canada

**Keywords:** User fees, Health policy, Universal health coverage, Perception, Experience, Burkina Faso

## Abstract

**Background:**

In 2016, the Gratuité policy was initiated by the Government of Burkina Faso to remove user fees for maternal, newborn, and child Health (MNCH) services. Since its inception, there has not been any systematic capture of experiences of stakeholders as it relates to the policy. Our objective was to understand the perceptions and experiences of stakeholders regarding the implementation of the Gratuité policy.

**Methods:**

We used key informant interviews (KIIs) and focus group discussions (FGDs) to engage national and sub-national stakeholders in the Centre and Hauts-Bassin regions. Participants included policymakers, civil servants, researchers, non-governmental organizations in charge of monitoring the policy, skilled health personnel, health facility managers, and women who used MNCH services before and after the policy implementation. Topic guides aided sessions, which were audio recorded and transcribed verbatim. A thematic analysis was used for data synthesis.

**Results:**

There were five key themes emerging. First, majority of stakeholders have a positive perception of the Gratuité policy. Its implementation approach is deemed to have strengths including government leadership, multi-stakeholder involvement, robust internal capacity, and external monitoring. However, collateral shortage of financial and human resources, misuse of services, delays in reimbursement, political instability and health system shocks were highlighted as concerns that compromise the government's objective of achieving universal health coverage (UHC). However, many beneficiaries were satisfied at the point of use of MNHC services, though Gratuité did not always mean free to the service users. Broadly, there was consensus that the Gratuité policy has contributed to improvements in health-seeking behavior, access, and utilization of services, especially for children. However, the reported higher utilization is leading to some perceived increased workload and altered health worker attitude.

**Conclusions:**

There is a general perception that the Gratuité policy is achieving what it set out to do, which is to increase access to care by removing financial barriers. While stakeholders recognized the intention and value of the Gratuité policy, and many beneficiaries were satisfied at the point of use, inefficiencies in its implementation undermines progress. As the country moves towards the goal of realizing UHC, reliable investment in the Gratuité policy is needed.

**Supplementary Information:**

The online version contains supplementary material available at 10.1186/s12961-023-01008-3.

## Background

There has been significant interest amongst governments in Africa to ensure that the entire population has access to good quality primary health care at an affordable cost. This focus on care access in Africa started since the Bamako Initiative, which was launched in 1987 [1]. The initiative was developed in the context of economic crises and the adverse effects of adjustment programs in many African countries. In response, experts proposed the total exemption of user fees for everyone or vulnerable persons who require essential health services [2].

In Burkina Faso, the first pilot projects for a user fee exemption scheme like had been proposed across the continent started in the late 2000s [3]. One of such schemes, which was first implemented between 2008 and 2015, was the Gratuité policy which was initially implemented as a non-governmental organization (NGO)-led user fee exemption pilot in districts of health regions of Sahel (Dori and Sebba), Boucle du Mouhoun (Tougan), and Nord (Séguénéga). During this pilot, NGOs subsidized 100% of the direct payment for the care received by pregnant women and children under-five years in all public health facilities [4, 5].

Following its adoption by the Council of Ministers of Burkina Faso on March 2, 2016 and driven by government’s political commitment to universal health coverage (UHC), the user fee exemption policy was run in three regions of the country from April 2 to May 31, 2016, and on June 1, 2016, Burkina Faso began implementing the Gratuité policy nationwide. Since then, the Gratuité policy has been and continues to be implemented in all public health facilities and some private facilities in the country. Public facilities provide a defined package of RMNCH services free of charge, fully funded by the government budget. Instead of charging out-of-pocket payments, equivalent fee-for-service payments are made to facilities by the central government. Funds were pre-positioned for the facilities initially every two months from 2016 to mid-2017 and then quarterly since the last quarter of 2017. Subsequent payments were adjusted based on service reports. To date, between 60 and 80% of the funds are earmarked for drugs, and facilities can use the remainder for services, such as consultations. The scheme is managed by the Ministry of Health and Public Hygiene (MoH & PH)’s Technical Secretariat for Universal Health Insurance Coverage and verification is contracted out to NGOs. The exemptions from the direct payment for essential health care services offered due to the policy will contribute to attaining the Sustainable Development Goals. The policy's long-term vision was to significantly reduce avoidable deaths among children aged 0–5 years and women [6, 7].

While there have been various research assessing the effectiveness of user fee exemption policies in Africa, only a handful have captured stakeholder perceptions and experiences of implementing such policies [8]. Such studies have also mostly focused on single stakeholder groups, for example, those published in Benin and South-Africa [9, 10]. After six years of implementation of the Gratuité policy in Burkina Faso, there is an opportunity to address this evidence gap. As such, our objective in this study was to understand the perceptions and experiences of multiple stakeholder groups regarding the implementation of the Gratuité policy in Burkina Faso. Such an evaluation is essential to capture lessons that can be helpful for the successful implementation of similar policies in the future.

## Methods

### Study design

This qualitative study was conducted over a period of six months, from the week commencing March 7, 2022. We reported the study findings following the 32-item consolidated criteria for reporting qualitative research (COREQ) [11] (Additional file 1).

### Study setting

The study covered national and regional stakeholders in the regions of Centre and Hauts-Bassin. Both regions were selected as they are both some of the most populated and most cosmopolitan regions in Burkina Faso. The regions are also inhabited persons across the socio-economic scale, from the poor many of whom live in the slums to the wealthy who live in the cities. Centre and Hauts-Bassin regions also hold districts that are the national capital and economic nerve center of the country respectively and are home to many of the stakeholders implicated in the policy. In addition, both are home to the two largest cities in the country, i.e., Ouagadougou and Bobo Dioulasso, respectively.

### Recruitment of study participants

Participants included policymakers, senior civil servants in the Ministry of Health, researchers, representatives of NGO responsible for monitoring the policy, skilled health personnel, health facility managers at different levels of the health system, and women who used maternity services before and after the implementation of the Gratuité policy. All participants were purposively sampled, with guidance on engagement informed by guidance from the International Association for Public Participation [12]. For this study, a list of stakeholders involved in implementing or evaluating the policy was drawn by staff of ThinkWell Institute in collaboration with staff of the MoH & PH’s Technical Secretariat. In drafting the list, we included key officials of the MoH & PH (policymakers and senior civil servants) who were directly involved in the implementation of the Gratuité policy and for the NGOs (Association Songui Manegré/Aide au Développement Endogène, Terre des Hommes, Save the Children, HELP, SOS Sahel, Réseau Accès aux Médicaments, Association la Voute Nubienne), we included the listed focal person based on the MoH & PH's database, For the health facilities, we reached out to facility leads of the largest public hospitals in the regions in the two regions (Centre Hospitalier Universitaire (CHU)-Yalgado Ouédraogo, CHU-Tengadogo, and CHU-Sourou Sanou) as well as one lower tier health facility each (Centre Medicale avec Antenne Chirurgicale (CMA) Bogodogo, CMA Kossodo (Centre)) and CSPS Samandéni (Hauts Bassins)). For completeness, we invited a private clinic in each region. The health facility leads nominated the skilled health personnel to be included in the study, ensuring a mix of doctors, midwives, and nurses). Prospective women who used maternity services before or after the implementation of the policy were identified to the research team by skilled health personnel in the selected health facilities across both regions.

For stakeholders invited to KIIs, such as officials of the MoH & PH, letters of invitation accompanied by informed consent were sent to them a minimum of three weeks before the proposed date. Personal contacts were also established either through email or telephone. Details on the purpose and proposed format of the interview (face-to-face or remote) were included in the invitation letter. Health workers invited to FGDs were recruited at their workplaces. Women who used maternity services were engaged when they used services in the selected health facilities or within the community working with the help of community leaders. As with other stakeholders, they received informed consent with the purpose and proposed format of the FGD. For all stakeholders, participation was entirely voluntary.

### Data collection

Collection of qualitative data was done through key informant interviews (KIIs) and focus group discussions (FGDs) with the sampled participants. Topic guides prepared in the French language and tailored to the various stakeholder groups seeking to understand issues that related to the implementation of the Gratuité policy were used to guide the KIIs and FGDs. For FGDs conducted with women, the topic guide was also developed in the local language, Douala. In all, three topic guides were developed including one for KIIs at the national and regional levels which targeted government and NGO representatives, one developed for the service providers at the facility level and another for the service users at the facility level. The topic guides were developed based on insights from a similar study that enquired about experience of implementing user fee exemption policies in Ghana and South-Africa [9, 10]. Across board, after establishing the purpose of the research, the topic guides focused on capturing some background details about the participant and their role/relationship as regards the policy, before progressing to collect data on their perception and experience on the implementation of the Gratuité policy, from the specific stakeholder perspective. For the topic guide used for national and regional level actors, the tool enquired about issues around perception of the policy, its implementation approach including perceived successes and failures, as well as challenges, resource allocation, disbursement mechanisms, stakeholder performance, impact of the policy on skilled health personnel and services, and recommendations for future design and programming of user fee exemption policies in Burkina Faso. In the tool for regional level actors, the tool was developed to place more emphasis on the process of translating policy implementation at the regional level. For the NGOs, additional questioning relating to their experience from monitoring the policy were asked. While the tool was also slightly modified to specifically focus on impact of policy on service delivery and utilization as well as experience of front-line policy implementation from the perspective of the health facility managers. Similar questions were asked in the topic guide for FGDs with skilled health personnel. For the women, the topic guide for the FGDs focused on understanding their experience of accessing and utilizing RMNCH services for themselves and their children under the age of 5 years before and after the implementation of the policy.

During the sessions (KIIs or FGDs), all participants were made to feel comfortable enough to express themselves and behave naturally (credibility) by establishing rapport. Our understanding of comments made by participants was repeated to them to verify that they conveyed their intended meaning (confirmability). Data collection continued until data saturation (i.e., the point at which new data repeat what was expressed in previous data) was achieved (dependability) [13–15]. Each FGD lasted between 60 and 90 min, and KII between 35 and 47 min. The topic guides were piloted before use for data collection. In all, we conducted four FGDs among 36 women who used maternity services before or after the implementation of the policy (two in each region), 2 FGDs with skilled health personnel (one in each region), and KIIs with 20 key stakeholders, including 15 KIIs conducted in the Centre region and five in the Haut-Bassin region (Table 1). The final sample size was deemed to have high information power based on the study objective, sample specificity involving different stakeholders at different levels (national and regional) and in two regions, the strong quality of the dialogue and data analysis from multiple points of view [13].

**Table 1:** Stakeholder groups with methods of engagement

Stakeholder group	Method of engagement	Number of sessions	Number of persons
Policymakers	KII	1 (Centre) 1 (Haut Bassin)	1 (Centre) 1 (Haut Bassin)
Senior civil servants	KII	5 (Centre) 2 (Haut Bassin)	5 (Centre) 2 (Haut Bassin)
NGO representatives	KII	7 (Centre)	7 (Centre)
Skilled health personnel	FGD	1 (Centre) 1 (Haut Bassin)	7 (Centre) 6 (Haut Bassin)
Health facility managers	KII	2 (Centre) 2 (Haut Bassin)	2 (Centre) 2 (Haut Bassin)
Women who used maternity services before or after the implementation of the policy	FGD	2 (Centre) 2 (Haut Bassin)	18 (Centre) 18 (Haut Bassin)

The KIIs and FGDs were conducted by two females (AMR and AG) and one make (AB-T), all of whom had postgraduate training experience (MSc. or PhD) in qualitative research at the time of the study and worked across academia and the development sector.

### Data analysis

All audio recordings were first transcribed verbatim in the original languages (French or Douala), with the resulting transcripts reviewed for accuracy. Following this, the FGDs in Douala were translated to French with back-translation done. A thematic analytic approach, which focuses on detecting and describing implicit and explicit ideas (themes) within the transcript, was applied for data reduction. A six-step approach involving data familiarization, initial code generation, searching for themes, reviewing themes, defining, and naming themes and producing the report was used in the study [16]**.**

For the data familiarization step, AB-T, AMR, and AG listened to the audio recordings and read through the transcripts multiple times to gain a comprehensive understanding of the data. In the initial code generation step, a deductive approach inspired by the interview guide was used to generate codes, while also allowing for open coding to capture any unanticipated themes [17]. In searching for themes, the researchers looked for patterns and connections among the codes, grouping them into preliminary themes. These themes were then reviewed and refined in the reviewing themes step, with the researchers discussing and debating their interpretations to ensure consistency and accuracy. In the defining and naming themes step, the researchers worked together to identify overarching themes that captured the essence of the data, defining them through detailed descriptions and examples, and giving them concise, informative names. Finally, in producing the report, the themes were organized and presented in a clear, logical manner, with quotes and examples from the transcripts used to support each theme. These analytical steps were performed with the aid of NVivo 12™ (QSR International, Memphis, USA). The text of the transcripts was analyzed as a proxy for the experience of the interviewees’ knowledge of the subject matter, perceptions, feelings, and behavior and interpreted while considering our interaction with the study participants during the KIIs and FGDs [18]. In this study, the research team included one member who was internal (insider—PY) with the rest being external (outsiders) to the policy [19]. As a team, we took a neutral position in analyzing and interpreting the data, ensuring that our previous opinions of the policy did not influence our analysis or interpretation of the findings emerging from the research [20]. AB-T, AMR, and AG (outsiders) who were not involved in the implementation of the policy conducted the analysis while all authors were involved in the interpretation of findings, with the PY mostly providing context for the interpretation.

## Findings

Five key themes emerged from the study and are presented below.

### Stakeholders have a positive perception of the Gratuité policy

There is recognition amongst majority of the stakeholders about what the policy was intended to achieve at inception. The intention was that all those who did not use services in the health centers for financial reasons would actually use such services if the financial barrier was addressed. This was a shared perception, irrespective of stakeholder group.

*“...the first objective of the policy was to break the financial barrier, which is one of the key barriers that needed to be broken to ensure people have access to health care” (Representative, NGO 3)*

*“...the Gratuité policy is to help the population, to improve the health of the population, to give wellbeing to children and women” (Participant 2 (P2) FGD, Samendeni)*

Some stakeholders stated that the government has a responsibility to support individuals to pay for their healthcare, more so for people who are relatively poor and amongst vulnerable populations such as women and children. Stakeholders pointed to the broad health system consequences if access to healthcare was not guaranteed for vulnerable populations with government agreeing that a user fee exemption policy such as Gratuité was a good one to support access to basic care.

*“…a really good strategy that allows vulnerable populations, including women and children, to have access to basic care” (UHC Technocrat, Ministry of Health and Public Hygiene (MoH & PH))*

The policy remains popular amongst the population, which was deemed to mean that government will find it difficult to even repeal the policy now. Indeed, several stakeholders agree that the policy remains valid today, as it did at inception. Many stakeholders talk about the need to sustain Gratuité but recognize that it is not a magic bullet. In this regard, stakeholders including government and NGO representatives pointed out that in addition to raising awareness about the policy and implementing other measures to reduce maternal and newborn mortality including recruiting and equipping skilled health personnel, ensuring functioning health facilities.

*“...any government that will suspend Gratuité will have difficulty convincing the population to get behind such a proposal because even those who complain that Gratuité is not good realize its importance when they have their child or their wife in the hospital” (Representative, NGO 4)*.

### The implementation approach has strengths, but concerns remain

Many stakeholders highlighted their general positive perception of the approach of implementing the policy. Some of the positive perceptions were related to the fact that the policy is financed by the government, the involvement of multiple stakeholders, the leadership and simplified coordination strategy of the technical secretariat, the strong capacity to distribute the funds, and the outsourcing of the monitoring function to NGOs.

*“In terms of strengths, it [Gratuité] is financed in by the state budget directly” (Representative, NGO 6)*

*“So, at the level of the technical secretariat, you can see that I am surrounded by researchers, NGOs and so on... I do not have everyone in my office. If I placed each one in the office, it would be a large structure. So, we instituted a mechanism. You do this, he or she does that, and so on. Then there is one person who is in the center to coordinate” (UHC Technocrat, MoH & PH)*

*“Another point that to be made is the fact of outsourcing the monitoring to the NGOs, which is even an innovation that allows for the separation of functions… The NGOs, being independent of the system, are thus able to make a good control to avoid having control of the costs” (Representative, NGO 5)*

One concern that was raised however was that there was no sufficient time given to the pilot led by the government and implemented over a sizeable period in the form the policy was designed to be implemented was highlighted by some stakeholders as a weakness of the intervention. This action was deemed a missed opportunity for lessons that could have been embedded in current implementation practice to maximize population gains.

*“...they did not take the time to conduct a pilot phase in one region for perhaps one year [led by the government], to collect any shortcomings and make corrections before extending it to the whole country.” (Representative, NGO 7)*

There is a scarcity of resources to fully finance the basket of services needed to institutionalize UHC fully, which is considered a significant threat to the policy. Concern was raised by some government and NGO representatives that the policy cannot continue to increase the services that are covered because of the scarcity of resources, especially when donor funding is not being used to support the policy.

*“...with the scarcity of resources if we continue and we do not put more money in the pot, it can be a threat” (UHC Technocrat, MoH & PH)*.

Already shortages in medicines are quite frequent, and it often causes discontent or misunderstanding among the beneficiaries in relation to health services. A government official said, *“the shortages of medicines are partly linked to the lack of financial resources because there are delays in payment”* (UHC Technocrat, MoH & PH). These delays are also causing enormous challenges for health facilities, with some considering closure *“…because they have several months of payment arrears as a result of Gratuité and this undermines the functioning of the health facilities”* (Representative, NGO 6). Even for the commodity supply that is currently available, some misuse was flagged with some stakeholders involved in verification, including double invoicing by health facilities and parents of children who do not qualify for care because of their age being pushed on the system to receive care.

*“There are parents who come with children who are six or seven years old and who insist that the child is five or four years old in order to benefit from the free service*. *We also see sometimes behaviors like double invoicing, you see a woman, for example, who receives Norplant this week and a few days later, we see another invoice exactly the same woman with insertion of Norplant” (Representative, NGO 7)*.

Different stakeholders raised the minimal increase in human resources for health available to deliver the policy despite the significant increase in service utilization. An NGO representative said, *“…whether the child has a cough or a small warm body, they go to the health centers, yet the number of health personnel has not increased.” (Representative, NGO 7)*. One health worker said, *“people come a lot, and there is not enough staff” (Head Nurse, CSPS Samandeni)*. Other stakeholders highlighted political instability *“…with changes of regime”* and *“…the crisis of COVID-19 which led to the closure of the borders of course”* as potential threats to the policy.

### Many satisfied clients at the point of use, but Gratuité does not always mean free

There was a generally high overall satisfaction with the policy among the women engaged as part of this evaluation. For example, the experience of several women in Samendeni while accessing care was mostly as intended – they reported that they got to the health facility and were able to receive care without paying out of pocket. One woman said regarding her experience accessing care for her child,

*“Recently he [her child] was sick, and we came, we didn't pay anything, we were given everything” (P4 FGD, Samendeni)*.

A representative of an NGO who does the verification in a number of regions also highlighted that there was high satisfaction with the policy, as evidenced by the satisfaction survey that they conduct within the community.

*“...we have seen a very, very high degree of satisfaction. In our region of the South-West, we have more than 78% of satisfaction, which is similar in the Center-East region.” (Representative, NGO 1)*.

However, some women raised some concerns, especially regarding Gratuité care not actually being *“free”* and only basic and relatively inexpensive medicines such as Paracetamol are being covered and, in some instances, no medicines are received by the women at point of care. Some women described the policy as not being *“effective”* and say they are not able to benefit from the free healthcare policy when they had needed it. One woman’s response in Kossodo described this experience saying,

*“We hear about Gratuité, but it is not totally effective. If we say that we really benefit from it, we are lying. For pregnant women like me, we were told that it is free, but when you arrive, you pay” (P5 FGD, Kossodo)*.

### Health-seeking behavior and utilization of services, especially amongst the most vulnerable, have improved

Various stakeholders believe it has helped to limit the hitherto inertia associated with seeking healthcare, leading to improved access and increased use of services, especially as people do not have to think about how much they will need to pay to access care. The policy is also deemed to have altered the health-seeking behavior of some individuals who typically seek traditional medicine providers because of minimal finances but are now seeking proper health facilities as well as amongst those who delay seeking care from a health facility.

*“If they don't have anything in their hand, even if they are sick, they don't want to go to a consultation, whether in a public or private health facility, because they think there is no point in trying. But now, with the Gratuité policy, it has really increased the rate of attendance of health facilities” (Health Facility Manager, Haut Bassin region)*.

*“Those who really didn't have the money to come now can access so in terms of attendance it has significantly improved attendance at the health facilities” (Skilled Health Personnel, Centre Médical avec Antenne chirurgicale (CMA) Bogodogo).*

Several women specifically stated that indeed, the policy has helped to alter their health-seeking behavior, especially when compared to the period preceding the launch of the policy. The policy has also been beneficial for women who had some money before seeking facility-based care but upon reaching care realized that they did not have enough.

*“...before Gratuité, access to health maternal and child health services was difficult. Even if you came with money for care, often the money was not enough, and you had to go back home to source more money before coming back for care. But now with Gratuité, it’s easier to access the health care service” (P6 FGD, Samendeni)*.

The policy is also deemed to have stimulated earlier presentation of children at health facilities which stakeholders have attributed to contributing to reduced child mortality. A health worker stated that *“…even for malaria, [before Gratuité], there was a lot of infant deaths because people were consulting late, but after the implementation of the policy, the deaths have decreased because now people often come early” (Head Nurse, Centre de Santé et de Promotion Sociale (CSPS) Samendeni)*. The perception of increased service utilization for children also appears to be backed up by data from verification exercises conducted by TdH staff and evidence gathered by the MoH & PH.

*“...a child would come once every two years before, but since Gratuité was put in place, children come at least three times a year now” (Representative, NGO 3)*

*“...you look at the evolution of the average number of new contacts per child and per year, and five or six years before the Gratuité policy, it was around 1.7 contact per child. And this indicator in 2016 has really experienced an increase until reaching 3.0 in 2017-2018.” (UHC Technocrat, MoH & PH)*

### Higher utilization is leading to some perceived increased workload and altered health worker attitude

There were concerns that the policy has led to increased workload for some health workers, reported by some skilled health personnel and health facility leads. For some, this was based on the premise that while there has been increased service utilization, the number of health workers employed has not increased. However, for government, while they recognize that there may be some increment in workload, they do not see it as a permanent feature of the scheme, describing it as a *“mixed picture”* where there is increased workload in some health facilities but nothing more than the norm in others.

*“It [Gratuité] has increased the workload because the number of health workers who were there has not increased” (Health Facility Manager, Centre region)*.

*“...we realized that there are indeed areas where the workload exceeded the norm, and there are areas where there was really no workload that exceeded the norm” (UHC Technocrat, MoH & PH)*.

In any case, this workload was perceived to be associated with a poorer attitude of health workers in providing care to clients, with one NGO representative saying,

*“I saw from the first days of the adoption of this policy how the health centers were crowded… Staff can find themselves treating several children a day. Naturally, at some point, they no longer have the attitude to be welcoming, to examine the children properly, to respect the treatment protocols, to really prescribe rationally and in a way that meets the needs of the children” (Representative, NGO 7)*.

From the side of the women, some did not feel that the attitude of health workers had changed in any negative way since the implementation of the Gratuité policy. NGO staff highlighted that the feedback forms submitted by women who used services in health centers as per of routine client satisfaction survey showed that in terms of reception, treatment, and confidentiality, they were generally well appreciated by the populations. However, some women in the FGDs reported that they experienced some form of disrespectful care which they attributed to the free care that was not experienced before the policy came into force.

## Discussion

In this study, we set out to understand perceptions and experiences of stakeholders regarding the implementation of the Gratuité policy till date. Generally, majority of the stakeholders had a positive perception of the Gratuité policy. This positive perception is similar to that reported for similar schemes in Ghana and South-Africa [9, 10]. However, like with other policies, there were issues of concern. Many beneficiaries engaged in our study were satisfied at the point of use, but Gratuité did not always mean free to service users at point of care. As per explanations highlighted by stakeholders engaged in this evaluation, they explained that this phenomenon is most likely multi-factorial, including because of a lack of medicines and supplies at the time patient’s presentation emanating from overuse of existing stock or lack of financial resources to replenish stock (stockout), rationing by health workers, or corrupt practices. Indeed, a previous survey showed that approximately 30% of women paid out-of-pocket for maternity services during the NGO-managed pilot scheme implemented in Burkina Faso [21]. This observation appears to be a common feature of user fee exemption policies in many low- and middle-income countries [22, 23].

There was consensus amongst stakeholders that the Gratuité policy has contributed to improvements in health-seeking behavior and utilization of services, especially amongst the most vulnerable. While many stakeholders, including women and skilled health personnel, specifically highlighted increased utilization for children, there was no suggestion that there was an increment in service utilization amongst women themselves. Indeed, it appears that the intervention incentivized parents to take their children for healthcare since they did not have to pay any fees for this service, with one NGO representative saying, *“…whether the child has a cough or a small warm body, they go to the health centers…”*. This corroborates findings from a recently published nationwide single arm interrupted time series on the Gratuité policy which had no comparison group, authors reported a 57% increase in rate of health facility visits in the month immediately following the policy’s launch [24]. In a previous study published in Ghana, a mixed picture of an influence of similar policy on maternal health service utilization was presented [9]. There have been some discussions about whether this increment is specifically due to the Gratuité policy. However, irrespective of whether the perceived increased utilization is due to the Gratuité policy or not, higher utilization was deemed to be leading to increased workload experienced by some health workers, which some stakeholders associated with reports of poorer staff attitude which may be compromising perceived quality of care. A similar policy in South Africa led to majority of health workers feeling burnout and considering giving up their jobs [10]. In our study, NGO representatives who monitor the policy may explain the altered attitude of health personnel toward patients. Many stakeholders in our study pointed to between no increment and only marginal increments in human resources of health available to execute the Gratuité policy in Burkina Faso. Indeed, recent global analysis shows that there has been only marginal increment in human resources for health in Burkina Faso with annualized rate of change averaging only 1% between 1990 and 2019 [25].

Government and non-government players considered government leadership, multi-stakeholder involvement, robust internal capacity, and external monitoring as strengths. However, the lack of a pilot phase led by the government before national scale-up was deemed a weakness by a few NGO representatives. This appears to be more about a *‘missed opportunity’* for lessons that could have been learnt to inform full policy implementation. However, NGO representatives highlighted a crucial opportunity to leverage as it relates to the recognition of the policy value amongst the population and global community. In contrast, threats such as shortage of financial and human resources, misuse of services, political instability and health system shocks were highlighted by stakeholders engaged in our study. As shown already in the literature, implementation of user fee exemption schemes is challenging, more so in an environment where insecurity is growing, and health workforce unrest undermines health service delivery [3, 8, 26]. For the Gratuité policy, in particular, there have been delays in the reimbursement of health facilities and disruptions in the supply of free medicines. These issues can potentially affect the policy gains, minimize value for money of investments made on the policy [27], and compromise the government's objective of realizing UHC.

### Implications for policy

There is a clear case for sustaining the Gratuité policy. At the very least, it allows parents to be more proactive about seeking care for their children. The simplest solution to the challenge of funding Gratuité will be to ensure the execution of this budget line. However, the Government also needs to fund several competing priorities, more so in a period of insecurity. The government could leverage the popular support for the Gratuité policy, as reported by stakeholders engaged in our study, to implement hypothecated or ring-fenced taxes for health. These have been implemented in other African countries to varying effectiveness in raising funds [28], and this could be an option for funding the Gratuité policy. However, stakeholders also recognized that the policy is not a magic bullet and needs to be complemented by other health system-strengthening interventions. This is in line with what has been reported in the literature [29, 30]. As such, collateral schemes such as quality improvement initiatives should be sustained if gains are to be maintained. In addition, one other key challenge of the policy reported as a threat relates to inadequate human resources. There is clear evidence that the cost of recruitment and retaining skilled health personnel is the major driver for MNCH service provision [31]. As such, responding to this challenge require exploration of innovative solutions to optimize policy gains. While evidence base on effectiveness of strategies in similar low-resource settings remains limited, strategies such as task-shifting to lower cadre health workers such as community health workers, provision of financial and non-financial incentives, and targeted recruitment, which have been implemented in high-resource settings may be considered [32, 33]. Furthermore, with stakeholders highlighting some corrupt practices, including double invoicing, and charging service users for MNCH services that should otherwise be free, there is a case for whistleblowing to allow service users raise complaints, been used in other settings and shown to be effective in reducing informal payments by health workers [34]. A realist evaluation of a user fee exemption policy in Benin showed that such bottom-up pressure aided policy compliance [35].

### Strengths and limitations

There are some strengths and limitations worth highlighting as regards this evaluation. First, this study included a broad range of stakeholders relevant to the policy. This is the first study that takes a multi-stakeholder perspective in evaluating the Gratuité policy, thereby providing a holistic stakeholder view of the intervention. Within each stakeholder group, there was also a good mix. This design allowed for the capture of varying views within stakeholder groups, which can aid future intervention/policy design [36]. In addition, the combination of KIIs and FGDs provided a breadth of data collection methods that allowed recruitment of all relevant stakeholders. A limitation is that the study was conducted in two regions only. Security challenges and limited resources prevented the conduct of the study in other regions. However, this limitation does not nullify the value of our study, as the study was conducted in the most populated and cosmopolitan regions in the country and we included national and regional level actors who work across and are conversant with the situation in several districts, including those in this evaluation. In any case, the number and diversity of KII and FGD participants, combined with the data saturation being reached, suggests that the findings would not necessarily be different if more participants were recruited. In addition, the study design included methods to guarantee trustworthiness such as using a standard protocol to guide conduct of the interviews and discussions, ensuring that participants were at ease to express themselves and verifying the intended meaning of the statements that they made.

## Conclusion

There is a general perception that the Gratuité policy is achieving what it set out to do, which is to increase access to care by removing financial barriers. While stakeholders recognized the intention and value of the Gratuité policy, and many beneficiaries were satisfied at the point of use, inefficiencies in its implementation undermines progress. As the country moves towards the goal of realizing UHC, reliable investment in the Gratuité policy is needed.

## Supplementary Information

**Additional file 1.** Completed consolidated criteria for reporting qualitative studies: 32-item checklist.

## Data Availability

Data generated in this study are available from the corresponding author on reasonable request.

## Abbreviations

CMA: Centre Médical avec Antenne chirurgicale
CSPS: Centre de Santé et de Promotion Sociale
FGD: Focus group discussion
KII: Key informant interview
MOH & PH: Ministry of Health and Public Hygiene
MNCH: Maternal, newborn, and child health
NGO: Nongovernmental organization
UHC: Universal Health Coverage
